# Impairments of Long-Term Synaptic Plasticity in the Hippocampus of Young Rats during the Latent Phase of the Lithium-Pilocarpine Model of Temporal Lobe Epilepsy

**DOI:** 10.3390/ijms222413355

**Published:** 2021-12-12

**Authors:** Tatyana Y. Postnikova, Georgy P. Diespirov, Dmitry V. Amakhin, Elizaveta N. Vylekzhanina, Elena B. Soboleva, Aleksey V. Zaitsev

**Affiliations:** Sechenov Institute of Evolutionary Physiology and Biochemistry of RAS, Saint Petersburg 194223, Russia; tapost2@mail.ru (T.Y.P.); diespirov.gp@yandex.ru (G.P.D.); dmitry.amakhin@gmail.com (D.V.A.); elizaveta.vyl@gmail.com (E.N.V.); soboleva.elena.1707@gmail.com (E.B.S.)

**Keywords:** astrocyte, D-serine, temporal lobe epilepsy, NMDA, field potential, long-term potentiation, hippocampus, glial fibrillary acidic protein, excitatory postsynaptic current

## Abstract

Status epilepticus (SE) causes persistent abnormalities in the functioning of neuronal networks, often resulting in worsening epileptic seizures. Many details of cellular and molecular mechanisms of seizure-induced changes are still unknown. The lithium–pilocarpine model of epilepsy in rats reproduces many features of human temporal lobe epilepsy. In this work, using the lithium–pilocarpine model in three-week-old rats, we examined the morphological and electrophysiological changes in the hippocampus within a week following pilocarpine-induced seizures. We found that almost a third of the neurons in the hippocampus and dentate gyrus died on the first day, but this was not accompanied by impaired synaptic plasticity at that time. A diminished long-term potentiation (LTP) was observed following three days, and the negative effect of SE on plasticity increased one week later, being accompanied by astrogliosis. The attenuation of LTP was caused by the weakening of N-methyl-D-aspartate receptor (NMDAR)-dependent signaling. NMDAR-current was more than two-fold weaker during high-frequency stimulation in the post-SE rats than in the control group. Application of glial transmitter D-serine, a coagonist of NMDARs, allows the enhancement of the NMDAR-dependent current and the restoration of LTP. These results suggest that the disorder of neuron–astrocyte interactions plays a critical role in the impairment of synaptic plasticity.

## 1. Introduction

Epilepsy is a common neurological disorder, significantly affecting patients’ quality of life [1]. One of the most frequent and poorly responsive to treatment forms of this disease is temporal lobe epilepsy (TLE). TLE affects multiple brain areas involved in acquiring and retaining memories, particularly the hippocampus [2,3,4]. Therefore, in adults with poor seizure control, cognitive decline may develop. Moreover, cognitive function maturation in children is highly susceptible to the adverse effects of epilepsy [5]. Therefore, understanding the molecular and cellular mechanisms underlying cognitive impairment in TLE is essential to developing effective therapies [6]. 

Cognitive weakening, including memory decline, is found in various animal models of TLE [7,8]. Among the multiple factors leading to cognitive impairment related to epilepsy, abnormalities in neuronal interaction’s molecular and synaptic mechanisms contribute most significantly [9,10]. The neural substrate for learning and memory is thought to be synaptic plasticity [11]. Depending on the pattern of synaptic input activation, there may be a prolonged increase or decrease in synaptic efficiency [12]. These phenomena are called long-term potentiation (LTP) [13] and long-term depression (LTD) [14], respectively. Alterations in synaptic plasticity following seizures are the most frequent result of model studies. However, the magnitude and direction of changes varied and depended on experimental conditions and the choice of an animal model. For example, acute pentylentetrazole-induced generalized seizures attenuated hippocampal LTP in rats 1, 3, and 7 days after seizures [15]. A decrease of LTP in latent [16,17] and chronic [18,19] phases was found using a lithium–pilocarpine model of epilepsy in rats. However, an enhancement of N-methyl-D-aspartate (NMDA) receptor-dependent LTP in the latent [19] and chronic [20,21] phases of this model was shown by other research groups in adult animals. 

Another critical factor that can cause cognitive impairment in epilepsy is neuronal loss and astrogliosis in the hippocampus and other areas of the temporal lobe [6,22]. Although numerous studies have shown that comparable seizures cause less damage in the immature brain compared to the mature brain [23,24], some studies indicate enhanced vulnerability of specific neuronal populations to experimental status epilepticus (SE) in young animals [25].

In the present study, we examined changes in the pharmacological properties of LTP during the first week following the epileptic status induced by pilocarpine in 3-week-old rats. The lithium–pilocarpine model is considered to be the most appropriate model of TLE [26,27] because it reproduces the main features of the pathological condition: (i) localization of seizure foci in the hippocampus, entorhinal cortex, or amygdala [28]; (ii) an “initial precipitating injury”, which often precedes the onset of TLE [4]; (iii) a latent period without seizures; and (iv) often hippocampal sclerosis [29]. We analyzed the quantitative characteristics of the neuronal loss and astrogliosis in the hippocampus of young rats during this period. We revealed that SE dramatically accelerates the programmed death of hippocampal neurons, which is also observed in control animals during this period of ontogenesis. In contrast to control animals, post-SE rats showed astrogliosis and attenuation of long-term synaptic plasticity in the hippocampus during the latent phase of the model. One of the mechanisms of plasticity impairment may be attenuation of NMDA receptor-mediated signaling.

## 2. Results

### 2.1. Pilocarpine-Induced SE Provokes Neuronal Loss in the Hippocampus of Young Rats

**Figure 1:**
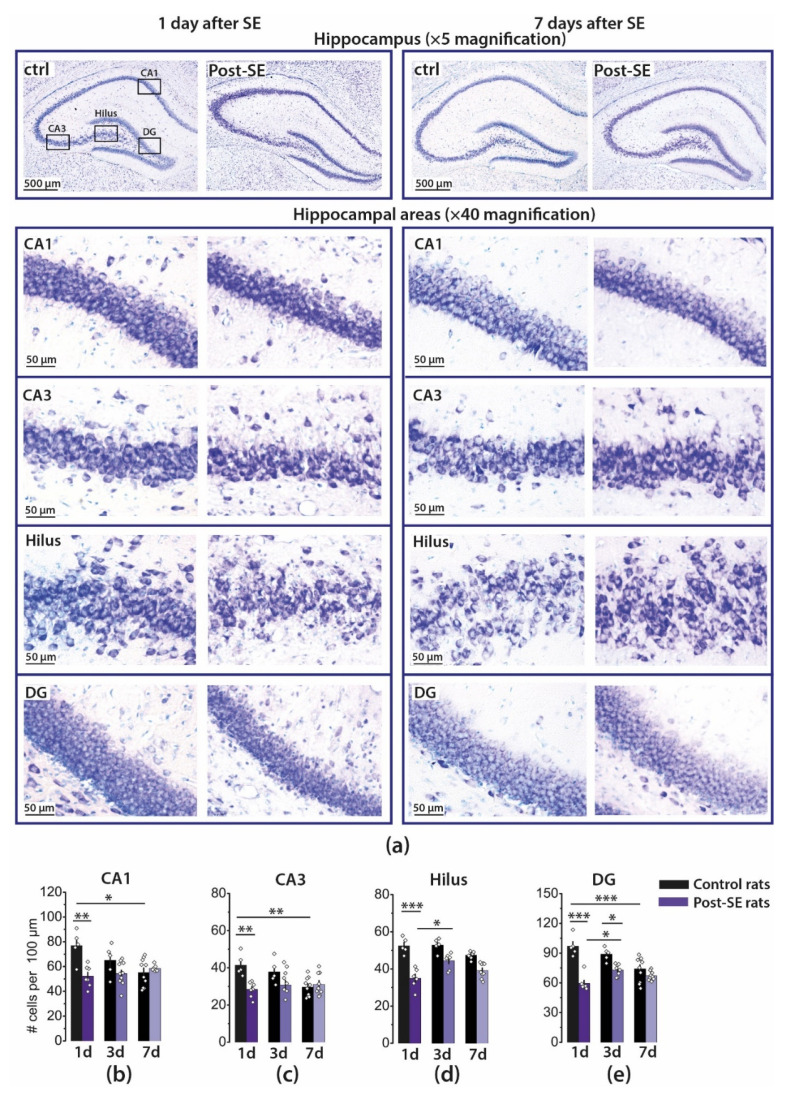
Pilocarpine-induced SE provokes neuronal loss in the hippocampus of young rats. (**a**) Representative sections of the Nissl staining of the different hippocampal areas CA1, CA3, hilus, and dentate gyrus (DG) in control (ctrl) and 7d post-SE rats. Group data of the counted Nissl-stained neurons per 100 μm in the hippocampal areas: CA1 (**b**), CA3 (**c**), hilus (**d**), and DG (**e**). Five to nine animals were in each group. The diamonds show the individual values for each rat. The bars indicate average values, and error bars show standard errors of the means. Two-way ANOVA was performed to determine the effects of SE, time after SE, and interaction of these factors: CA1 (SE: F_1, 39_ = 12.5, *p* < 0.01; time: F_2, 39_ = 2.2; *p* = 0.13; SE × time: F_2, 39_ = 7.3, *p* < 0.01); CA3 (SE: F_1, 38_ = 9.1, *p* < 0.01; time: F_2, 38_ = 3.7; *p* < 0.05; SE × time: F_2, 38_ = 5.8, *p* < 0.01); hilus (SE: F_1, 34_ = 79, *p* < 0.001; time: F_2, 34_ = 7.2; *p* < 0.01; SE × time: F_2, 34_ = 5.3, *p* < 0.01); DG (SE: F_1, 38_ = 58, *p* < 0.001; time: F_2, 38_ = 6.4; *p* < 0.01; SE × time: F_2, 38_ = 12, *p* < 0.001). * *p* < 0.05, ** *p* < 0.01, *** *p* < 0.001 (Tukey’s post hoc test).

First, we analyzed which regions of the hippocampus are most vulnerable to pilocarpine-induced status epilepticus and when the maximum neuronal death occurs. Using Nissl-stained brain sections (Figure 1a), we counted the neurons in the pyramidal layers of the CA1 and CA3 areas of the hippocampus, hilus, and granular cell layer of the DG at 1, 3, and 7 days after SE (1d, 3d, 7d post-SE groups) and in appropriate controls (Figure 1b–e). 

A two-way ANOVA revealed a significant effect of SE, time after SE, and the interaction of these factors on the number of neurons in each area. Tukey’s post hoc tests revealed significant differences in the number of neurons in control and 1d post-SE rats for all examined regions and DG in 3d post-SE rats. The number of neurons decreased approximately by one-third in all examined areas. This result corresponds well with the data obtained in the lithium–pilocarpine model in two- and three-week-old rats in the previous studies [25,30]. 

However, the neuronal death that occurred a day after SE subsequently becomes undetectable due to the developmental decrease in the number of neurons. We noticed a significant decline in the number of neurons in CA1, CA3, and DG in the hippocampi of control animals within a week. In contrast, in post-SE rats, the number of neurons did not decrease in these areas within the same period (Figure 1). Because of this, no significant differences between control and 7d post-SE rats in the number of neurons were found, and the results obtained one week after SE are consistent with the data that prolonged seizures do not cause a significant neuronal loss in the hippocampus in early life [23,31]. 

Thus, we suggest that seizures significantly accelerate developmental neuronal loss in the hippocampus of young animals and probably alter this process, leading to persistent deleterious effects.

### 2.2. Astrogliosis in the Rat Hippocampus Develops by the End of the First Week after SE

Status epilepticus leads to an increase in the number of astrocytes and changes in astrocyte expression profile as well as in their morphology, biochemistry, and functionality, a process called reactive astrogliosis [32]. Previous studies have shown that the first signs of astrocyte activation can occur following seizures as early as one day after seizures in the lithium–pilocarpine model [33]. 

However, astrocyte-specific marker glial fibrillary acidic protein (GFAP) production in the hippocampus of adult rats does not change in the first five days after SE [33], and, in young rats, an increase in GFAP production is observed two weeks after pilocarpine-induced SE [18,34]. Therefore, we analyzed astrocyte activation by counting the GFAP immunofluorescence area using a densitometric method. According to a two-way ANOVA, a significant effect of SE on the GFAP-positive area was revealed in all hippocampal regions studied. 

**Figure 2:**
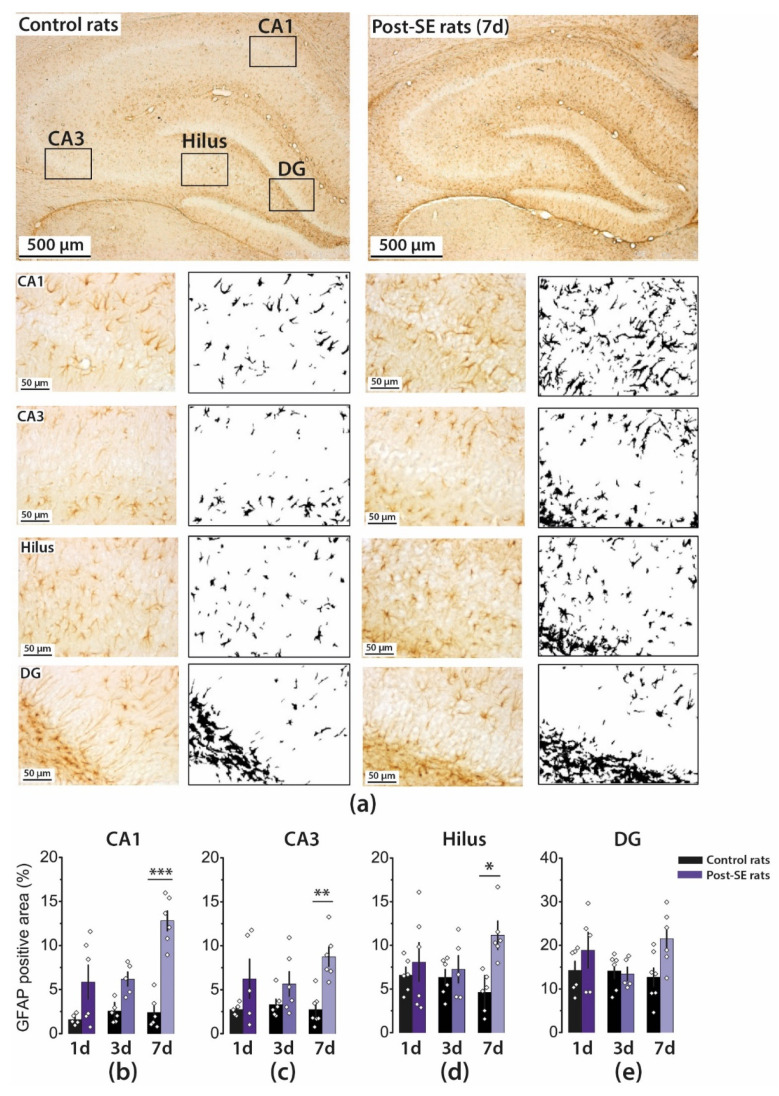
Astrogliosis in the rat hippocampus following pilocarpine-induced SE. (**a**) Immunohistochemistry targeting glial fibrillary acidic protein (GFAP) was used for the detection of astrocytes. Representative images of hippocampal sections with GFAP-positive cells and their corresponding black–white masks, which were obtained with ImageJ software and used for area counting (upper panels: ×5 magnification and lower panels: ×40 magnification). The averaged GFAP-positive areas in different hippocampal regions: CA1 (**b**), CA3 (**c**), hilus (**d**), and DG (**e**). Five to seven animals were in each group. The diamonds show the individual values for each rat. The columns indicate average values, and error bars show standard errors of the means. Two-way ANOVA was performed to determine the effects of SE, time after SE, and the interaction of these factors: CA1 (SE: F_1, 28_ = 48, *p* < 0.001; time: F_2, 28_ = 7.6; *p* < 0.01; SE × time: F_2, 28_ = 6.1, *p* < 0.01); CA3 (SE: F_1, 29_ = 17.4, *p* < 0.001; time: F_2, 29_ = 0.82; *p* = 0.45; SE × time: F_2, 29_ = 1.4, *p* = 0.26); hilus (SE: F_1, 29_ = 7.3, *p* < 0.05; time: F_2, 29_ = 0.32; *p* = 0.73; SE × time: F_2, 29_ = 2.7, *p* = 0.09); DG (SE: F_1, 29_ = 4.6, *p* < 0.05; time: F_2, 29_ = 1.1; *p* = 0.36; SE × time: F_2, 29_ = 2.0, *p* = 0.16). * *p* <0.05, ** *p* < 0.01, *** *p* < 0.001 (Tukey’s post hoc test).

**Figure A1:**
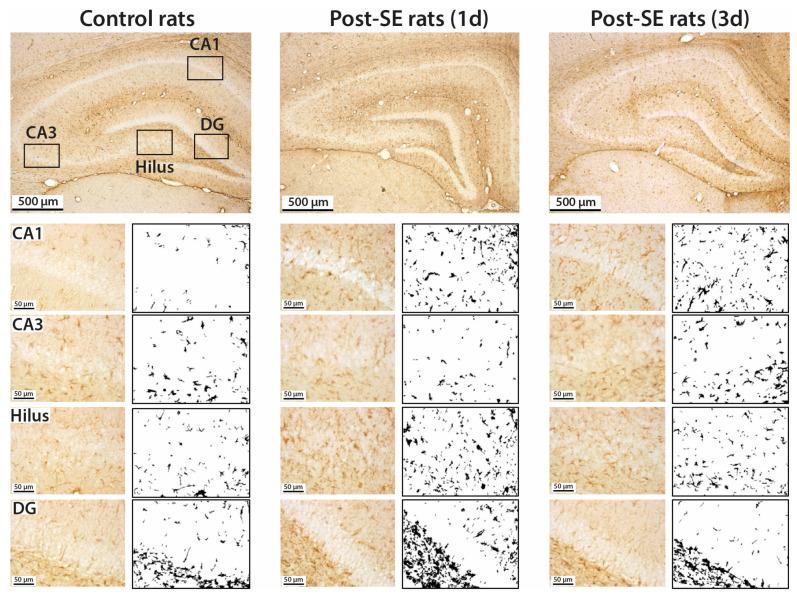
Astrogliosis in the rat hippocampus following pilocarpine-induced SE. Immunohistochemistry targeting glial fibrillary acidic protein (GFAP) was used for the detection of astrocytes. Representative images of hippocampal sections with GFAP-positive cells and their corresponding black–white masks were obtained with ImageJ software and used for area counting (upper panels: × 5 magnification and lower panels: × 40 magnification).

In the group of post-SE rats, the GFAP-positive area increased by Day 7, whereas in control rats, it remained constant (Figure 2 and Figure A1). Therefore, the most significant differences between the groups were found on Day 7. Astrogliosis was most pronounced in the CA1 region; the area increased fivefold from 2.3 ± 0.8 to 12.8 ± 1.1%, whereas in the DG, astrogliosis was less prominent (13 ± 2% and 22 ± 3%).

We also observed alterations in the distribution of astrocytes in the hippocampal layers in the post-SE group. The difference with controls was most pronounced in the CA1 area. Astrocytes migrate to *stratum pyramidale*, and their processes may overlap.

### 2.3. LTP Is Attenuated in the CA1 Hippocampal Area in Post-SE Rats

**Figure 3:**
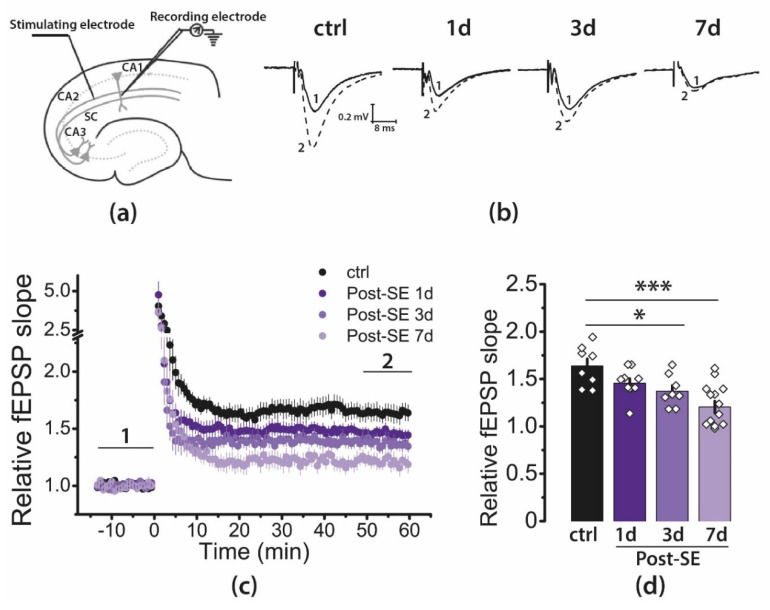
LTP is attenuated in the CA1 hippocampal area in post-SE rats. (**a**) Schema showing the positions of electrodes in the hippocampus. (**b**) Representative examples of fEPSP were recorded before induction (1) and 50–60 min after HFS (2). (**c**) Diagram showing the normalized slope of fEPSP in control (ctrl) and post-SE groups (1d; 3d; 7d). (**d**) Bar diagram illustrates differences in LTP value between groups. Seven to ten animals were in each group. One or two brain slices were used from one animal. The diamonds show the individual values for each brain slice. All data are presented as a mean ± standard error of the mean. One-way ANOVA F_3, 32_ = 8.85, *p* < 0.001; Tukey’s post hoc test reveals the significant differences with control group: * *p* < 0.05, *** *p* < 0.001.

Next, we examined LTP at CA3-CA1 synapses in acute hippocampal brain slices from control and post-SE rats (Figure 3). LTP was measured 1, 3, and 7 days following pilocarpine-induced SE. Control rats were tested one day after saline injection. High-frequency stimulation (HFS) resulted in robust LTP in hippocampal CA1 neurons of control rats (1.64 ± 0.07, *n* = 8). According to one-way ANOVA, the post-SE rats showed significantly reduced LTP compared with control rats (F_3, 32_ = 8.9, *p* < 0.001, Figure 3d). Tukey’s post hoc tests revealed no difference in LTP level in the control and 1d post-SE groups (1.46 ± 0.05, *n* = 10, *p* > 0.05). However, LTP was significantly decreased 3 and 7 days after SE (3d post-SE: 1.37 ± 0.06, *n* = 8, *p* < 0.05; 7d post-SE: 1.20 ± 0.06, *n* = 10, *p* < 0.001). 

### 2.4. Pharmacological Properties of LTP Changed during the Latent Period of the Lithium–Pilocarpine Model in Young Rats

LTP induction in CA3-CA1 synapses of the hippocampus is an NMDAR-dependent process [12,13]. A variety of synaptic plasticity abnormalities have been identified in seizure models [16,17,18,19,21]. Thus, the weakening of LTP after SE may be caused by the disruption of the molecular mechanisms of LTP production.

**Figure 4:**
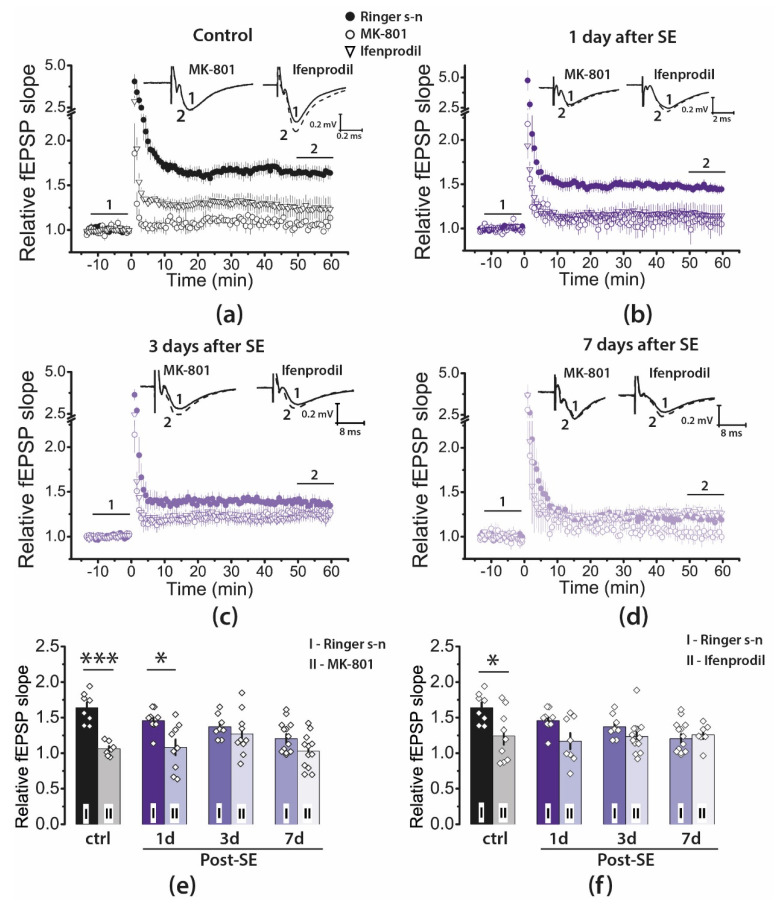
Pharmacological properties of LTP in the CA1 hippocampus of juvenile rats during the latent phase of the lithium–pilocarpine model of temporal lobe epilepsy. (**a**–**d**) The normalized fEPSP slope in the control (**a**) and experimental groups (**b**–**d**) in the presence of the NMDAR blocker MK-801 (10 μM) or ifenprodil (3 μM), a selective GluN2B-containing NMDAR antagonist before and after HFS. Representative examples of fEPSP were recorded before induction (1) and 50–60 min after HFS (2). (**e**,**f**) diagrams illustrating the magnitude of plasticity in the control and experimental groups in the presence of MK-801 (**e**) or ifenprodil (**f**). Between 7 and 10 animals were in each group. One or two brain slices were used from one animal. The diamonds show the individual values for each brain slice. Two-way ANOVA following Tukey’s post hoc tests was used. For simplicity, only the effects of the drugs are marked in the figure: * *p* < 0.05, *** *p* < 0.001.

To determine whether the NMDAR-dependent mechanism of LTP induction persisted after SE, we induced LTP in the presence of MK-801 (10 μM), an uncompetitive NMDAR antagonist (Figure 4). Two-way ANOVA revealed a significant effect of MK-801 application (F_1, 66_ = 33, *p* < 0.001) as well as effects of SE (F_3, 66_ = 4.1, *p* < 0.01) and a combination of these factors (F_3, 66_ = 3,7, *p* < 0.05). In control, MK-801 application prevented LTP induction (Figure 4a,e; *n* = 7). In post-SE rats, MK-801 significantly reduced LTP magnitude in the 1d post-SE group (*n* = 9). However, in two other post-SE groups, the effect did not reach the level of statistical significance, most likely because the LTP magnitude was low even without applying the blocker (Figure 4b–e, Post-SE 3d: *n* = 10; 7d: *n* = 12). Thus, the results of this experiment are consistent with the persistence of the NMDAR-dependent mechanism of LTP induction.

Since our previous studies indicate that SE alters the subunit composition of NMDARs [35,36,37], we investigated the effect of ifenprodil (3 μM), a selective antagonist of GluN2B-containing NMDARs (Figure 4a–d,f). Application of ifenprodil significantly affected LTP induction (two-way ANOVA: F_1, 66_ = 11.9, *p* < 0.01), and the effect of ifenprodil differs between groups (F_3, 66_ = 2.9, *p* < 0.05, Figure 4f). Ifenprodil reduced the magnitude of LTP only in the control group (*n* = 9) but did not affect it in any of the post-SE groups (1d: *n* = 8; 3d: *n* = 14; 7d: *n* = 7). 

**Figure 5:**
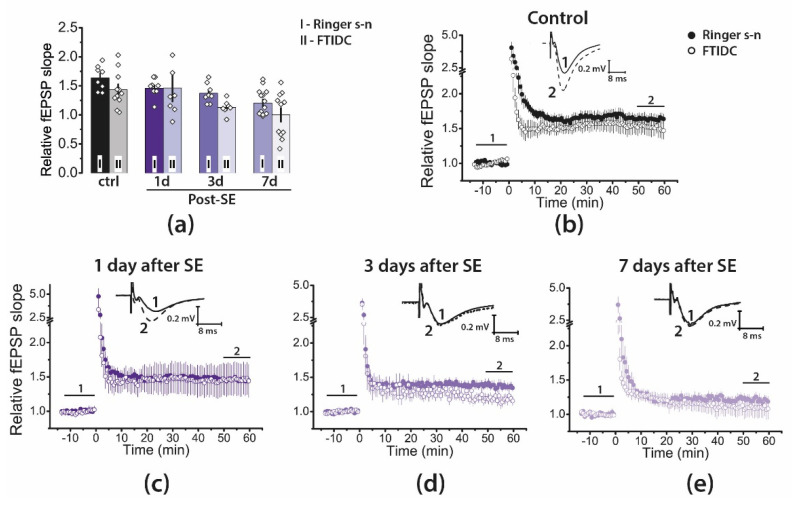
Pharmacological properties of LTP in the CA1 hippocampus of juvenile rats during the latent phase of the lithium–pilocarpine model of temporal lobe epilepsy. (**a**) Diagram illustrating the magnitude of plasticity in the control and experimental groups in the presence of FTIDC. Between 7 and 10 animals were in each group. One or two brain slices were used from one animal. The diamonds show the individual values for each brain slice. Two-way ANOVA following Tukey’s post hoc tests was used. No significant effects of FTIDC were detected. (**b**–**e**) The normalized fEPSP slope in the control (**b**) and experimental groups (**c**–**e**) in the presence of a selective mGluR1 antagonist, FTIDC (5 μM), before and after HFS. Representative examples of fEPSP in the presence of FTIDC were recorded before induction (1) and 50–60 min after HFS (2).

The dysfunction of metabotropic glutamate receptors can cause impaired plasticity. Group I mGluRs are located on the postsynaptic membrane perisynaptically [38], are involved in the induction and maintenance of LTP [39], and also have a modulating effect on NMDARs [40,41]. Our previous study revealed that pentylenetetrazole-induced SE suppressed NMDA-dependent LTP, and LTP transiently switched to the mGluR1-dependent form [42]. Therefore, we also tested the effect of a selective mGluR1 antagonist, FTIDC (5 μM), on LTP induction (Figure 5). FTIDC application did not significantly affect LTP induction in any group (two-way ANOVA: F_1, 65_ = 2.5, *p* = 0.12; control: *n* = 11; Post-SE groups—1d: *n* = 7; 3d: *n* = 7; 7d: *n* = 11). These results suggest that LTP induction in CA3-CA1 synapses does not require the activations of group I mGluRs.

**Figure 6:**
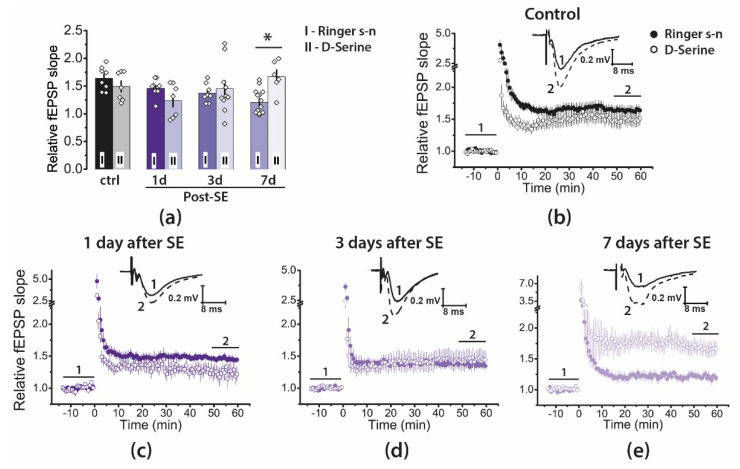
D-serine, a coagonist of NMDARs, enhances LTP in the 7d post-SE group. (**a**) Diagrams illustrating the magnitude of plasticity in the control and experimental groups in the presence of D-serine. Between 7 and 10 animals were in each group. One or two brain slices were used from one animal. The diamonds show the individual values for each slice. Two-way ANOVA following Tukey’s post hoc tests was used. For simplicity, only the effect of D-serine is marked in the figure: * *p* < 0.05. (**b**–**e**) The normalized fEPSP slopes in the control and post-SE groups in Ringer’s solution and the presence of D-serine (10 μM) before and after HFS. Representative examples of fEPSP in presence of D-serine were recorded before induction (1) and 50–60 min after HFS (2).

Recently, we have shown that D-serine application fully restored the initial phase of LTP (5–15 min) in the hippocampus of the rat during the chronic stage of the lithium–pilocarpine model, although exogenous D-serine was ineffective for a later phase of LTP (50–60 min) [18]. In the present study, we tested the effect of D-serine (10 μM) on LTP during the latent phase of the lithium–pilocarpine model (Figure 6). 

We found that the effect of D-serine on synaptic plasticity differed between groups (two-way ANOVA: F_3, 58_ = 4.9, *p* < 0.01; control: *n* = 7; Post-SE groups—1d: *n* = 7; 3d: *n* = 10; 7d: *n* = 6). We found a significant increase in LTP magnitude only in the 7d post-SE group. In this group, the LTP value under the application of D-serine was restored to the control level.

### 2.5. SE Affects the Properties of NMDAR-Mediated Synaptic Currents 

**Figure 7:**
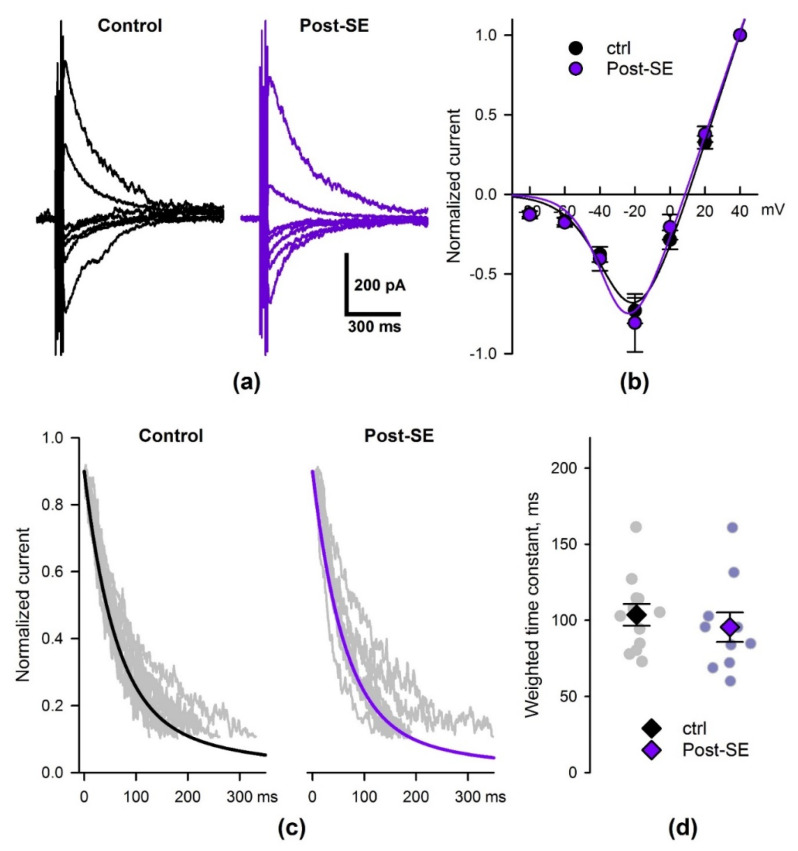
The properties of NMDAR-mediated eEPSC are not altered in 7d post-SE rats. (**a**) A representative set of NMDAR-mediated eEPSCs, recorded at different holding voltages (from +40 to –80 mV, 20 mV increments). (**b**) The average I–V relationships, obtained from the control and 7d post-SE groups and fitted with Equation 1 (see Methods). No differences between control (*n* = 12) and 7d post-SE (*n* = 10) groups were detected for all parameters (*V*_12_: –29 ± 2 vs. –31 ± 3 mV; *k*: 10.3 ± 0.8 vs. 8.4 ± 1.6; *V_rev_*: 9.8 ± 1.4 vs. 7.5 ± 2 mV, respectively; *t*-test, *p* > 0.05 for all three comparisons). (**c**) The decays of NMDAR-mediated eEPSCs from the control and post-SE groups. Gray traces represent the superimposed raw experimental recordings. The solid black and purple lines represent the double exponential functions (Equation 2) with the average parameters corresponding to the control and post-SE groups. (**d**) The weighted time constants, obtained for the control and post-SE groups. No significant difference was detected (*t*-test, *p* = 0.5).

As found in our previous study [43], the impairment of synaptic plasticity was accompanied by the weakening of NMDAR-mediated synaptic currents in hippocampal pyramidal neurons. Therefore, we investigated the seizure-induced changes in NMDAR-mediated currents using the whole-cell patch-clamp registration in the 7d post-SE group (Figure 7). The eEPSCs were recorded in the presence of gabazine (10 μM) and DNQX (10 μM). 

First, we investigated if the shape of the NMDAR current–voltage relationship (I–V relationship) was altered in the post-SE animals. The NMDAR-mediated currents were induced by five 100-Hz stimuli to reduce variability in the response amplitude. Responses were recorded at different holding voltages from +40 to –80 mV (Figure 7a). The peak values of eEPSCs were normalized to the current amplitude at +40 mV (Figure 7b). The resulting curves were fitted using Equation (1) (Methods). No significant differences between the control and post-SE groups in parameters of this equation were detected. This result indicates that the voltage dependence of NMDARs is not affected by SE.

The increased weighted decay time constant of NMDAR-current was observed 1–3 days following pilocarpine-induced SE [44]. Thus, next, we investigated whether the kinetics of NMDAR-mediated current were still altered in the 7d post-SE group. The 90−10% decay phase of eEPSC recorded at –40 mV was fitted by two exponential functions (Equations (2) and (3)) with the time constants of 60 and 300 ms [43,44]; weighted time constants were determined (Figure 7d). No significant differences between the weighted time constants in control and post-SE groups were detected (*t*-test, *p* = 0.5). Thus, the kinetics of NMDAR-mediated eEPSCs were unaltered in the 7d post-SE group. 

Implementation of HFS results in prolonged glutamate release. Therefore, the NMDAR desensitization or the rate of glutamate clearance may strongly affect the postsynaptic response [45]. Thus, next, we investigated the properties of NMDAR-mediated currents elicited by HFS in the control and 7d post-SE groups; we also tested the effect of D-serine in the 7d post-SE group (post-SE + D-Ser). 

As HFS induces long-term plasticity of NMDA-receptor-mediated synaptic transmission in the hippocampus [46,47], only a single implementation of this protocol was performed per slice. The stimulus strength was selected so that the peak current value in response to five 100-Hz stimuli was about –250 pA at –35 mV. No significant difference between the stimulation current strength in the three experimental groups was observed (control: *n* = 10, 400 ± 40 µA; post-SE: *n* = 11, 410 ± 60 µA; post-SE + D-Ser: *n* = 11, 350 ± 30 µA; one-way ANOVA: F_2,31_ = 0.48, *p* = 0.6).

**Figure 8:**
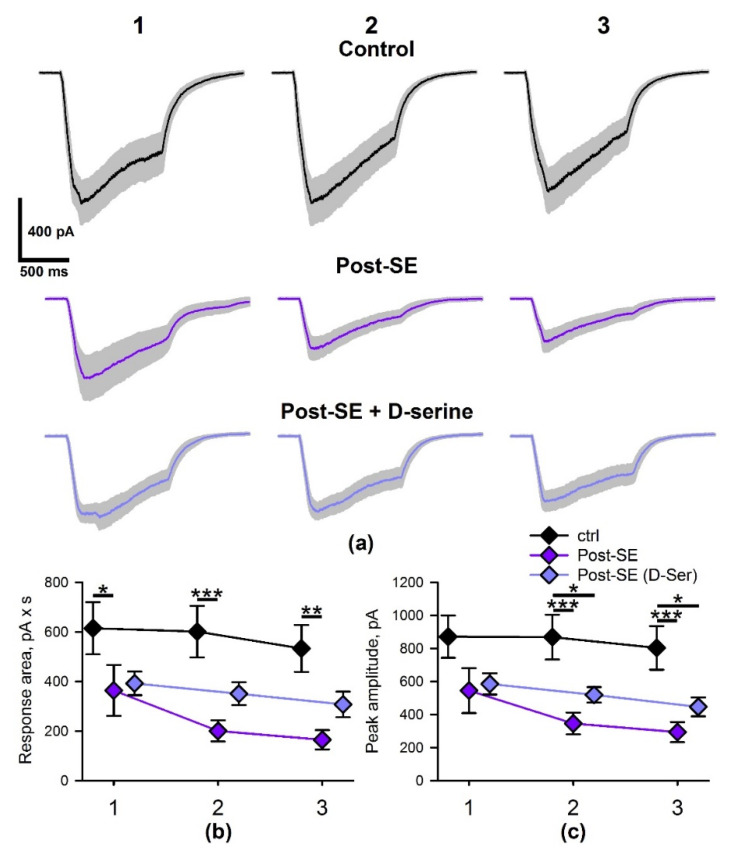
The magnitude of NMDAR-mediated currents evoked by HFS is reduced in post-SE rats. (**a**) The average voltage-clamp recordings of NMDAR-mediated currents, induced by HFS in CA1 neurons in the control (upper trace, *n* = 10) and post-SE rats without (middle trace, *n* = 11) and with (lower trace, *n* = 11) D-serine. Gray areas represent the standard error of the mean. According to the mixed-design ANOVA, the average areas (**b**) and peak amplitude (**c**) of NMDAR-mediated currents in the three groups are significantly different. Asterisks indicate the significant difference between the values, corresponding to the same stimulus train number (Tukey’s test): * *p* < 0.05; ** *p* < 0.01; *** *p* < 0.001.

Trains of 100 stimuli at 100 Hz induced strong NMDAR-mediated currents, whose amplitude increased during the first 7–24 stimuli, followed by a gradual decrease after that (Figure 8a). 

We performed a mixed-design ANOVA analysis of NMDAR-mediated current area and peak amplitude in three groups. The analysis revealed that the response areas and amplitudes are significantly different in these groups (Figure 8b,c; F_2, 95_ = 6.6, *p* < 0.01, and F_2, 95_ = 7.0, *p* < 0.01 for areas and amplitudes, respectively), with the effect strength being independent of the stimulus train number (F_4, 95_ = 1.24, *p* = 0.3 and F_4, 95_ = 1.20, *p* = 0.3 for areas and peaks, respectively). The post hoc tests demonstrated that the response areas and amplitudes were significantly lower in the post-SE group compared to the control. D-serine partly restored the response area, as no significant differences between post-SE + D-Ser group with the other two groups were detected. 

Thus, NMDAR-mediated currents in hippocampal cells during HFS are significantly lower in the post-SE group than in the age-matched control animals. The bath application of D-serine can partly compensate for the decrease in NMDAR-mediated current.

## 3. Discussion

**Figure 9:**
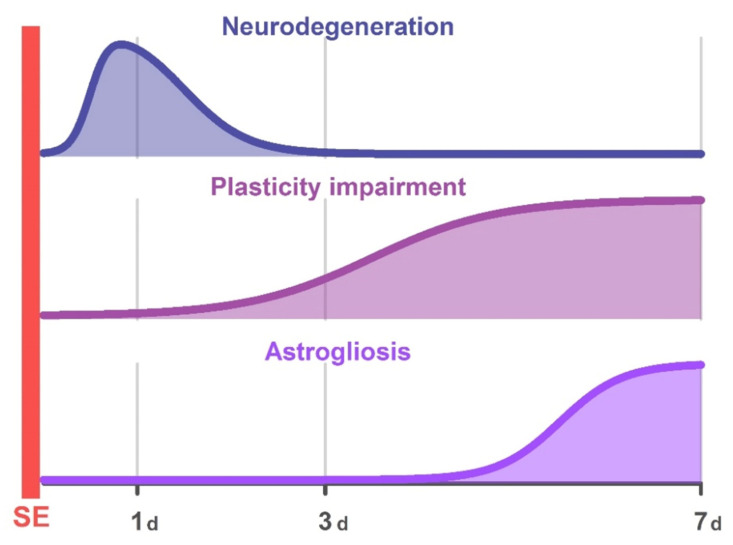
Main changes in the immature rat hippocampus followed pilocarpine-induced SE. The shaded area indicates periods when the differences between the control and post-SE groups were statistically significant. Abbreviations: SE—status epilepticus, d—day after SE.

In this study, we examined the morphological and electrophysiological changes in the hippocampus of young rats after SE induced by pilocarpine over a week. Figure 9 summarizes the study’s main results on the changes induced by SE.

The earliest effect of seizures is a rapid neuronal death induced by an excitotoxic effect of glutamate. We found that nearly one-third of neurons in the hippocampus and dentate gyrus died on the first day, agreeing with the previous report [25]. However, one week later, no differences in the number of hippocampal neurons between the control and post-SE groups were detectable. Therefore, we assume that SE accelerates the death of neurons that should have undergone age-related elimination.

We found no abnormalities in synaptic plasticity on the first day after SE. However, a decrease in LTP was observed following three days, and the negative effect of SE on plasticity increased after one week and was accompanied by astrogliosis. The attenuation of plasticity is caused by the weakening of NMDAR-dependent signaling. In particular, the magnitude of NMDAR-dependent current decreased more than two-fold under the HFS protocol in the group of post-SE rats. Probably, one of the reasons for NMDAR-dependent signaling attenuation is a disorder of neuron–astrocyte interactions. Application of glial transmitter D-serine, a coagonist of NMDARs, allows enhancing the NMDAR-dependent current during the application of HFS protocol to a significant extent and restoring LTP.

The weakening of LTP found in this study is consistent with most experimental data obtained in various SE models [16,17,18,19]. Many molecular mechanisms have been identified that lead to LTP attenuation [16,42,48,49]. One hypothesis suggests that the potentiation of synaptic transmission occurred due to epileptic activity, and classical LTP have a common mechanism of implementation and NMDAR dependence [48,50,51,52], so further synaptic potentiation after seizures is weakened as a result of occlusion. For example, transient insertion of calcium-permeable GluA2-lacking AMPARs is necessary for LTP consolidation in hippocampal synapses, and blockade of this subtype of AMPARs impairs LTP expression [53]. Pilocarpine-induced SE also resulted in a transient increase in AMPARs-mediated neurotransmission supported by enhanced levels of calcium-permeable GluA1-containing AMPARs [50,54]. 

A recent study suggests that alterations in the levels of phosphorylation/dephosphorylation at the carboxy-terminal domain of different AMPAR subunits may play an essential role in impaired plasticity [16] since the levels of phosphorylation/dephosphorylation modulate AMPAR trafficking and long-term synaptic plasticity [55,56]. 

In this work, we focused on another molecular mechanism, specifically NMDAR-mediated signaling during LTP induction. Epileptic activity changes the number of NMDARs [56] and their functional properties, which depend directly on their subunit composition [57]. Previously, several studies using pilocarpine and lithium–pilocarpine models of epilepsy in rats demonstrated an increase in the relative contribution of GluN2B-containing NMDA receptors in the early stages of epileptogenesis [44,49,58]; similar results were obtained in the model with pentylentetrazole kindling [59]. It has also been shown that increased phosphorylation of the GluN2B subunit of NMDARs can result from seizures [60]. 

Genetic and pharmacological studies have shown that synaptic GluN2A-containing NMDARs play a major role in inducing LTP. In contrast, extrasynaptic GluN2B-containing NMDARs play a significant role in causing long-term depression [57,61,62,63,64,65,66]. Therefore, it has been hypothesized that the ratio of GluN2A and GluN2B subunit levels is the most significant factor in determining the sign of synaptic plasticity [67], and changing their ratio affects plasticity. 

We examined the effect of ifenprodil, a specific GluN2B-containing NMDA receptor antagonist, on synaptic plasticity, but found no significant difference in the impact of ifenprodil in the control and post-SE groups. In addition, we compared the properties of NMDAR-mediated currents in the control and 7d post-SE groups, where plasticity was maximally reduced. We revealed that voltage dependence and the decay kinetics of NMDAR-mediated eEPSCs were unaltered in the 7d post-SE group. Since a change in the GluN2B/GluN2A ratio affects the decay kinetics of responses [44], and an increase in the proportion of GluN2D-containing NMDARs additionally alters voltage dependence [68], our result suggests that there are no changes in the subunit composition of the NMDARs in the 7d post-SE group. Thus, LTP impairments in the latent phase (one week after SE) cannot be explained by changes in the subunit composition of NMDARs.

However, we found a significant difference between the NMDAR-mediated currents induced by HFS in the control and 7d post-SE rats. Each burst of HFS results in a one-second-long period of synaptic activity, and the area and amplitude of responses were reduced in 7d post-SE group. The high-affinity glial and neuronal excitatory amino acid transporters (EAATs) could effectively clear glutamate released during 100 Hz stimulation [69]. However, glutamate clearance could slow down following the bursts of high-frequency activity [70]. Moreover, the relationships between astrocytic leaflets expressing EAAT2 and neuronal synaptic elements are highly dynamic [71]. In epileptic tissue, the glutamate clearance could be further disturbed due to alterations in EAAT functional activity [72] and decreased astrocytic surface area at glutamatergic synapses [18,73,74]. 

Thus, during the prolonged synaptic activity, the mechanisms of glutamate clearance might be overwhelmed in the post-SE group, leading to more pronounced desensitization of NMDARs than in the control group. The desensitization generally occurs due to receptor accumulation in long-lived closed states [45,75]. Statistical models suggest that NMDARs can desensitize by switching either to states in which the glutamate and coagonist (glycine or serine) are bound to the receptor or to states in which the coagonist molecules are not bound to the receptor [76]. The latter process results in the glycine-dependent desensitization of NMDAR macroscopic current [76]. The binding of glutamate to GluN2 reduces the affinity of GluN1 for glycine through negative allosteric modulation; this leads to a gradual decrease in ionic current through NMDAR [77]. An increase in glycine concentration abolishes this effect [78].

In our preparation, D-serine restored the NMDAR-dependent LTP and enhanced the HFS-induced NMDAR-mediated current in post-SE rats, suggesting that glycine-dependent desensitization is augmented following seizures. Indeed, glycine and D-serine availability may be impaired in post-SE groups. For example, increased expression of glycine transporter 1 (GlyT1) has been detected in epileptic tissue, suggesting dysfunctional glycine signaling in epilepsy [79,80]. Thus, the dynamics of glycine concentration in the hippocampus during the prolonged stimulation can be altered, which in combination with possible alterations of glutamate concentration dynamics can account for the observed decrease of NMDAR-mediated currents. 

The decrease in available D-serine may be due to disturbances in neuron–glial relations. It was previously shown that hippocampal astrocytes retain the ability to control LTP within or near their individual territories, involving Ca^2+^-dependent D-serine release [81]. In this work, we have revealed that the CA1 region of the hippocampus shows the most pronounced increase in the GFAP-positive area. Previously, it was found that astrogliosis is accompanied by a change in the morphology of astrocytes, in particular, by a decrease in the number of astrocytic leaflets located in the immediate vicinity of the synapse [18]. This can also disrupt the local D-serine supply to specific NMDAR populations and affect their desensitization. Thus, the results of this study support the hypothesis that neuron–glia interaction in epileptogenesis is impaired, and the particular aspects of neuron–glia interaction require further investigation.

## 4. Materials and Methods

### 4.1. Animals and the Lithium–Pilocarpine Model of Temporal Lobe Epilepsy

Male Wistar rats were used in this study. At P20, rats from the same litters were randomly separated into control and pilocarpine groups to avoid possible genetic differences. All experiments were approved by the Sechenov Institute of Evolutionary Physiology and Biochemistry Ethics Committee. 

On P20, animals were injected with LiCl (127 mg/kg; Sigma-Aldrich, St. Louis, MO, USA). Status epilepticus was induced on P21 with intraperitoneal pilocarpine (30 mg/kg; Sigma-Aldrich). Peripheral cholinergic effects were reduced by pretreatment with the muscarinic acetylcholine receptor antagonist methylscopolamine (0.5 mg/kg; Sigma-Aldrich), administered 40 min before pilocarpine treatment injection. Age-matched control animals received LiCl and methylscopolamine, but saline was administered instead of pilocarpine. 

Animals were video-monitored for 2 h. Between 20 and 40 min after pilocarpine injection, about 90% of the animals developed generalized convulsions. The intensity of seizures was assessed with the modified Racine limbic seizures scale: (1) mouth and facial movement, (2) head nodding, (3) forelimb clonus, (4) rearing with forelimb clonus, and (5) rearing and falling with forelimb clonus [7,82]. Only animals that exhibited scale 3–4 seizures for at least 90 min (status epilepticus [SE]) were taken further into the electrophysiological experiments. To increase survival rate, rats were administered with a 5% glucose solution 2 and 24 h after pilocarpine injection. Experiments were performed 1, 3, or 7 days after pilocarpine-induced SE, which corresponds to the acute and latent phases of epilepsy in this model [26,27]. 

### 4.2. Hippocampal Slice Preparation

Control and post-SE rats were decapitated, and brains were quickly removed and cooled by ACSF (0 °C) containing in mM: 126 NaCl, 24 NaHCO_3_, 2.5 KCl, 2 CaCl_2_, 1.25 NaH_2_PO_4_, 1 MgSO_4_, and 10 glucose and saturated with a carbogen (95% O_2_ and 5% CO_2_). Horizontal slices (400 μm) containing dorsal hippocampus were cut using a vibratome (HM 650V; Microm International, Walldorf, Germany). After cutting, the slices were left in oxygenated ACSF for 1 h at 35 °C.

### 4.3. Field Potential Recordings and LTP Induction

The recordings were performed as previously described [15,42,43]. Briefly, the fEPSPs were registered from the stratum radiatum of the CA1 with the glass electrodes (0.2–1.0 MΩ). Synaptic responses were evoked by stimulation of the Schaffer collaterals using a bipolar nichrome electrode. The stimuli (duration—0.1 ms) were delivered every 20 s via an A365 stimulus isolator (World Precision Instruments, Sarasota, FL, USA). The value of the stimulation current was adjusted to elicit a response with a magnitude of 40–50% of maximal and was then fixed at this level.

Responses were amplified by a Model 1800 amplifier (A-M Systems, Carlsborg, WA, USA), then digitized with ADC/DAC NI USB-6211 (National Instruments, Austin, TX, USA) using WinWCP v5.x.x software (University of Strathclyde, Glasgow, UK). The electrophysiological recordings were analyzed using Clampfit 10.2 (Axon Instruments, San Jose, CA, USA). 

Once unchanging fEPSPs were obtained for 20–25 min (baseline), LTP was induced by HFS (3 trains every 20 s consisting of 100,100-Hz pulses). LTP was quantified by calculating the ratio of the average slope of the potentiated fEPSPs (50–60 min after HFS) and the baseline fEPSPs (10 min before HFS). 

**Figure A2:**
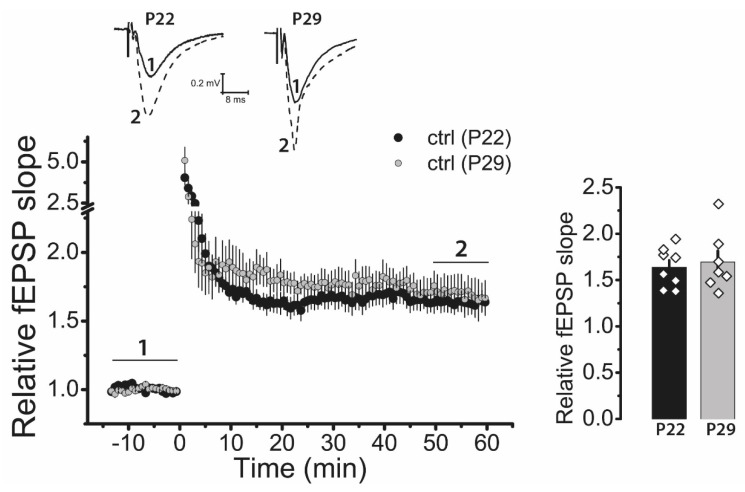
LTP values in the CA1 hippocampal area are the same in control rats at ages P22 and P29. Representative examples of fEPSP were recorded before induction (1) and 50–60 min after HFS (2). The diagram on the left is showing the normalized slope of fEPSP in control rats of two ages. The bar diagram illustrates no differences in LTP value between groups (*t*-test = 0.41, *p* = 0.70; P22: *n* = 8; P29: *n* = 7) The diamonds show the individual values for each brain slice. Data are presented as a mean ± standard error of the mean.

In preliminary study, we compared LTP properties 1 and 7 d after saline injection (22- and 29-d-old, respectively). No difference was found between these two groups (Figure A2). Therefore, we compared LTP properties in the post-SE rats with those in the 22-d-old control group.

### 4.4. The Whole-Cell Patch-Clamp Recordings

The pyramidal neurons were visualized using a Zeiss Axioskop 2 microscope (Zeiss, Oberkochen, Germany), equipped with differential interference contrast optics and a video camera (Grasshopper 3 GS3-U3-23S6M-C; FLIR Integrated Imaging Solutions Inc., Wilsonville, OR, USA). Patch electrodes (3–4 MΩ) were pulled with a P-1000 pipette puller (Sutter Instrument, Novato, CA, USA) from borosilicate glass capillaries (Sutter Instrument, Novato, CA, USA). The whole-cell voltage-clamp recordings were performed using a cesium methanesulfonate-based pipette solution (composition in mM: 127 CsMeSO_3_, 10 NaCl, 5 EGTA, 10 HEPES, 6 QX314, 4 ATP-Mg, and 0.3 GTP; pH adjusted to 7.25 with CsOH). 

The MultiClamp 700B (Molecular Devices, Sunnyvale, CA, USA) patch-clamp amplifier, NI USB-6343 A/D converter (National Instruments, Austin, TX, USA) and WinWCP 5 software (University of Strathclyde, Glasgow, UK) were utilized. The data were filtered at 5 kHz and sampled at 25 kHz. Access resistance was less than 15 MΩ and remained stable (≤20% increase) across the experiment in all cells included in the analysis. The liquid junction potential was compensated offline by subtracting 7 mV from the value of the holding voltage.

The I–V relationships of NMDAR-mediated currents were approximated using the following equation [83]:

(1) $$I_{NMDA}\left(V\right)=\frac{g_{inf}}{1+\exp\left(\frac{V_{12}-V}{k}\right)}\left(V-V_{rev}\right)$$

where $g_{inf}$ is the receptor conductance without the Mg^2+^ block as *V* approaches infinity; $V_{12}$ and k determine the voltage dependence of the Mg^2+^ block of NMDARs; and $V_{rev}$ is the reversal potential of the current. The main variable is marked in bold font.

The time course of the NMDAR-mediated eEPSC was described using the non-linear regression analysis of its 90–10% decay phase [43,44]. The biexponential function was utilized to fit the decays:

(2) $$I\left(t;A_{fast},\tau_{fast},A_{slow},\tau_{slow}\right)=A_{fast}*\exp\left(-\frac{t}{\tau_{fast}}\right)+A_{slow}*\exp\left(-\frac{t}{\tau_{slow}}\right)$$

where $A_{fast}$ and $\tau_{fast}$ are the amplitude and time constant of the fast-decaying component; $A_{slow}$ and $\tau_{slow}$ are the amplitude and time constant of the slow-decaying component. The $\tau_{fat}$ and $\tau_{slow}$ were set equal to 60 and 300 ms, respectively, during the approximation.

The weighted time constant was estimated using the following equation:

(3) $$\tau_{weighted}=\frac{\tau_{fast}*\;A_{fast}+\tau_{slow}*A_{slow}}{A_{fast}+A_{slow}}$$

### 4.5. Histology

#### 4.5.1. Tissue Preparation

Rats were anesthetized with Xylazine (50 µL per 100 g) and Zoletil (6 mg per 100 g) mixed in a water solution (1 mL per 100 g). Next, rats were perfused transcardially with phosphate-buffered saline (PBS, pH 7.4, 0.01 M) followed by 4% paraformaldehyde in PBS. After perfusion, the brain was removed and fixed by the 4% paraformaldehyde solution for 2–7 days at 4 °C. Next, brains were cryoprotected with 30% sucrose and stored at −80 °C. The 20-μm-thick frontal serial sections (from −2.6 to −3.6 mm to the bregma) were cut on a cryostat Bright OTF5000 (Bright Instrument Co Ltd., Huntingdon, UK). 

#### 4.5.2. Nissl Staining

Nissl staining was performed as previously described [43]. Sections were analyzed using the Leica Microscope AF 7000 (Leica Microsystems, Wetzlar, Germany) under ×400 magnification. For morphological analysis, neuronal counts were performed on every fifth section (yielding 8–10 sections from one rat hippocampus). The distance between the analyzed sections was 100 µm. The number of neurons in digital micrographs was counted per 100 μm for the cell layer in CA1, CA3, hilus, and dentate gyrus using ImageJ (U. S. National Institutes of Health, Bethesda, MD, USA).

#### 4.5.3. Immunohistochemistry

The distribution of the glial marker GFAP was analyzed using indirect immunofluorescence analysis. First, sections were treated with 3% H_2_O_2_ for 30 min to block endogenous peroxidases activity. After that, sections were rinsed in PBS 3 × 10 min and incubated with 0.2% TritonX-100 (Merck, Darmstadt, Germany) in PBS for 30 min. Next, sections were incubated in blocking serum (3% normal goat serum; 2% bovine serum albumin; 0.2% Triton X-100 in PBS) for 2 h. Then specimens were incubated in blocking serum with the primary mouse antibody against GFAP (1:1000; cat # NBP1-05197, Bio-Techne Ltd., Abingdon, OX14 3NB, UK) overnight at 37 °C. For DAB staining, sections were incubated with a biotinylated goat anti-mouse secondary antibody (1:500 in PBS, cat # BA-9200-1.5, Vector Laboratories Inc, Burlingame, CA, USA) at room temperature for 1 h. A streptavidin–biotin detection system was used. DAB-stained slices were examined using Leica Microscope AF 7000 (Leica Microsystems, Germany), and the images were analyzed using ImageJ.

The software converts all immunolabeled elements that fall within a threshold range into black pixels, and the rest of the image is converted into white pixels (Figure 2). Next, we calculated the percentage of black and white pixels. Finally, the percentages were used for the comparison between the groups using a two-way ANOVA.

### 4.6. Statistical Analysis

The statistical analysis and graphical representation of the results were performed using OriginPro 8 (OriginLab Corporation, Northampton, MA, USA), Statistica 8.0 (Systat Software Inc., Palo Alto, CA, USA), and Sigmaplot 12.5 (Systat Software Inc., San Jose, CA, USA). Dixon’s *Q* test (at a 90% confidence level) was used to identify and reject outliers. The normality of the sample data was evaluated using the Kolmogorov–Smirnov test. Statistical significance was assessed using Student’s *t*-test and one-way or repeated-measures ANOVA as stated in the text. All data are presented as mean ± standard error of the mean. *p* < 0.05 was considered statistically significant.

## Data Availability

The data presented in this study are available on request from the corresponding author.
